# Molecular characterisation of *NPM1* and *FLT3-ITD* mutations in a central South African adult *de novo* acute myeloid leukaemia cohort

**DOI:** 10.4102/ajlm.v10i1.1363

**Published:** 2021-06-30

**Authors:** Jean F. Kloppers, André de Kock, Johané Cronjé, Anne-Cecilia van Marle

**Affiliations:** 1 Department of Haematology and Cell Biology, School of Pathology, Faculty of Health Sciences, University of the Free State, Bloemfontein, South Africa; 2 Department of Haematology and Cell Biology, Universitas Academic Unit, National Health Laboratory Services, Bloemfontein, South Africa

**Keywords:** acute myeloid leukaemia, AML, *NPM1*, *FLT3-*ITD, frequency, South Africa

## Abstract

**Background:**

Recognition of molecular abnormalities in acute myeloid leukaemia (AML) has improved our understanding of its biology. *NPM1* and *FLT3-*ITD mutations are recurrent in AML and clinically significant. *NPM1* mutations are associated with a favourable prognosis, while *FLT3-*ITD mutations are an independent poor prognostic factor in AML.

**Objective:**

This study described the prevalence and molecular characteristics of the *NPM1* and *FLT3-*ITD mutations in a newly diagnosed AML patient cohort in central South Africa.

**Methods:**

The study included 40 de novo AML patients. An *NPM1* and *FLT3-*ITD multiplex polymerase chain reaction assay was optimised to screen patients for the respective mutations and were confirmed using Sanger sequencing. The prevalence of the *NPM1* and *FLT3-*ITD mutations were determined, and mutation-specific characteristics were described in relation to patients’ demographic information and AML classifications.

**Results:**

The patients’ median age was 38.5 years, with 77.5% (*n* = 31) of patients being self-proclaimed Black Africans. AML with recurrent genetic abnormalities was most prevalent (57.5%; *n* = 23), of which acute promyelocytic leukaemia (APL) was most common (40.0%; *n* = 16). None of the patients had the *NPM1* mutation. *FLT3-*ITD was present in 37.5% (6/16) of APL patients and in one (20.0%) of five AML patients with a t(8;21) translocation. Most patients had an *FLT3-*ITD allele ratio of ≥ 50% and ITD lengths of > 39 bp.

**Conclusion:**

*FLT3-*ITD mutations were mainly found in APL cases at a similar prevalence as reported in the literature. High *FLT3-*ITD allele ratios and long ITD lengths predominated. No *NPM1* mutations were detected.

## Introduction

Over the past decade, the field of acute myeloid leukaemia (AML) diagnostics has shifted from a primarily clinicopathological assessment to an integrated approach, including morphology, immunophenotyping, cytogenetics, and molecular genetics.^1,2^ In an era of precision medicine, the use of molecular genetic data, in particular, has fundamentally shaped the diagnostic approach to patients with AML.^3^ Genetic profiling has led to more accurate diagnostic classification and improved risk stratification and identification of potential therapeutic targets.^1^ Acute myeloid leukaemia is a genetically heterogeneous disease.^2^ Recurrent structural chromosomal aberrations detected by conventional cytogenetics were previously considered the most relevant prognostic variable in addition to the patient’s age and performance status.^2,3^ Three main cytogenetic risk groups (low, intermediate, and high) were defined, with approximately 50% of patients having a normal karyotype and falling into the intermediate cytogenetic risk group.^3,4^

The recognition of additional molecular abnormalities, especially in cytogenetically normal AML (CN-AML), has helped to improve our understanding of the disease biology and better predict outcomes.^3,4^ Mutational analysis performed on diagnostic samples of AML patients enrolled in the Eastern Cooperative Oncology Group E1900 trial demonstrated somatic mutations in as many as 97.3% of these patients.^5^ The Cancer Genome Atlas Research Group^6^ has shown that, on average, patients with AML have 13 acquired mutations at the time of diagnosis across the entire genome. Two genes in which recurrent mutations, which often co-occur, with known clinical significance have been identified in patients with AML include the gene encoding nucleophosmin protein-1 (*NPM1*) and one of the signalling genes, *FMS* (Feline McDonough Sarcoma gene)-related tyrosine kinase 3 (*FLT3*).^3,6^ *NPM1* mutations are associated with a favourable prognosis,^3^ while the *FLT3*-internal tandem duplication (ITD) mutation is considered an independent poor prognostic factor in patients with AML.^7^ However, the co-occurrence of these two gene mutations modulates their prognostic impacts.^3,7^

Nucleophosmin is a nucleolar phosphoprotein that shuttles between the nucleus and the cytoplasm.^3^ Nucleophosmin protein-1 is thought to be involved in many cellular functions, including protein synthesis, DNA replication, and regulation of the cell cycle.^3^ The reported frequency of *NPM1* mutations in patients with AML varies between 27% and 30% and is considered the most frequently identified molecular abnormality in CN-AML, occurring in 45% – 60% of cases.^4,6,8,9^ Four common mutations cause mutant *NPM1* (one 4 bp duplication and three separate 4 bp insertions). These mutations are all restricted to exon 12, the region that encodes the C-terminus of *NPM-1*, and result in a frame shift.^10^ The *FLT3* gene encodes a Class III receptor tyrosine kinase. Mutations in signalling genes such as *FLT3*, which lead to constitutive activation of the receptor tyrosine kinase, confer survival and proliferative advantage to cells.^3,9^ *FMS*-related tyrosine kinase 3 mutations occur in approximately one-third of patients with AML, and the ITD mutation is considered the second most common molecular abnormality in CN-AML (28% – 34% of cases).^4,9^ *FMS*-related tyrosine kinase 3-ITD mutations usually occur between exons 14 and 15 and can range between 3 bp and more than 400 bp in size.^11^

The limited reports on the prevalence of the *FLT3*-ITD and *NPM1* mutations in African AML populations indicate lower frequencies than reported internationally. In an Egyptian study that included 123 AML patients, *FLT3*-ITD and *NPM1* mutations were detected in 17.9% and 19.5% of patients.^12^ Another study by Sofan et al. (2014) detected *NPM1* mutations in 28% of CN-AML.^13^ Contrary to these findings, no *NPM1* mutations were found in a study of 100 Sudanese AML patients.^14^ The frequency of *NPM1* and *FLT3*-ITD mutations in a South African adult de novo AML cohort was 7.5% and 12%.^15^

This study investigated the presence and molecular characteristics of the *NPM1* and *FLT3*-ITD mutations in a newly diagnosed AML population in central South Africa. The study further aimed to describe the *NPM1* and *FLT3-*ITD mutations in relation to patient demographics and specific AML classifications.

## Methods

### Ethical considerations

Approval for the study was obtained from the Health Sciences Research Ethics Committee from the University of the Free State (study approval number: UFS-HSD2018/1174/2711) and the Free State Province Department of Health (study approval number: FS_201810_016). Written informed consent was obtained from all participants prior to sample collection. Samples were allocated study numbers to ensure patient confidentiality, and patient data were stored on password-protected devices, which were only accessible to the researchers.

### Study population

All adult patients (*n* = 40; coded FN1–FN40) that were diagnosed with de novo AML at the Universitas Academic Hospital in Bloemfontein, South Africa, during the study period (November 2018 – December 2019), and who were able to give written informed consent were included in this study. All AML patients were included regardless of cytogenetic findings. Patients’ demographic variables (age, sex, and self-proclaimed ethnic background) and disease-specific characteristics (AML classification according to the World Health Organization’s *Classification of Tumours of Haemopoietic and Lymphoid Tissues*, revised 4th edition^16^) were obtained from patients’ medical records. Based on the laboratory information records that were reviewed, none of the patients had any previous clonal myeloid disorder. Blood samples were obtained from each participant for this study during routine blood collection by the treating physician.

### DNA extraction

Genomic DNA was extracted from whole blood samples using the Wizard^®^ Genomic DNA Purification Kit (Promega, Madison, Wisconsin, United States). DNA samples were quantified using the BioDrop™ µLITE instrument (BioDrop, Cambridge, United Kingdom).

### *NPM1* and *FLT3-*ITD multiplex PCR optimisation

Anonymised samples that were positive and negative for the *NPM1* and *FLT3*-ITD mutations and formed part of an external quality control programme (generously donated by the Department of Haematology at Charlotte Maxeke Johannesburg Academic Hospital, National Health Laboratory Service, Johannesburg, South Africa) were used to optimise the assay. Published primers, fluorescently labelled with hexadecimal colour and fluorescein amidites fluorophores^17^ were used in the assay (*NPM1* Forward: 5’- GTT TCT TTT TTT TTT TTT CCA GGC TAT TCA AG- 3’; Reverse: 5’- HEX CAC GGT AGG GAA AGT TCT CAC TCT GC- 3’; and *FLT3*-ITD Forward: 5’FAM- AGCA ATT TAG GTA TGA AAG CCA GCTA- 3’; Reverse: 5’- CTT TCA GCA TTT TGA CGG CAA CC- 3’). Primers were evaluated for target-specificity using the Basic Local Alignment Search Tool (available at https://blast.ncbi.nlm.nih.gov/Blast.cgi) from the National Center for Biotechnology Information (NCBI, Bethesda, Maryland, United States).

A polymerase chain reaction (PCR) mixture was prepared with the GoTaq® DNA polymerase (Promega Madison, Wisconsin, United States) reagents. Components of the kit were manually mixed and the mixture consisted of 5 *µ*l 5Х GoTaq Buffer, 2 *µ*L of MgCl_2_, 0.5 *µ*L of dNTPs, and 0.2 *µ*L of GoTaq DNA polymerase (5 U/*µ*L). Additionally, 1 *µ*L of genomic DNA (100 ng/*µ*L), 14.3 *µ*L of nuclease-free water, and 1 *µ*L each of the forward and reverse primers (5 *µ*M) (for *FLT3* and *NPM1*) were added to the PCR mixture. A temperature gradient experiment was used to determine the optimal PCR annealing temperature for the primers. The gradient temperatures ranged between 65 °C and 70 °C. The subsequent PCR cycling conditions were one cycle at 95 °C for 10 min, 45 cycles of 95 °C for 20 s, 65 °C for 40 s, and 72 °C for 40 s, and a final step of one cycle at 72 °C for 32 min. Polymerase chain reaction products were subjected to capillary electrophoresis (3500 Genetic Analyser, Applied Biosystems, Foster City, California, United States). Briefly, 1 *µ*L of each sample was mixed with 9.5 *µ*L of Hi-Di™ Formamide (Applied Biosystems, Foster City, California, United States) and 0.5 *µ*L GeneScan™ 600 LIZ™ size standard (Applied Biosystems, Foster City, California, United States). The optimal primer concentrations (5 *µ*M) and DNA limit of detection were determined as part of the optimisation procedure. The *NPM1* and *FLT3*-ITD multiplex PCR assay had a lower limit of detection of 0.8 ng/*µ*L. Capillary electrophoretic results were analysed using GeneMapper version 6 software (Applied Biosystems, Foster City, California, United States).

### *NPM1* and *FLT3-*ITD detection in acute myeloid leukaemia patients

**FIGURE 1:**
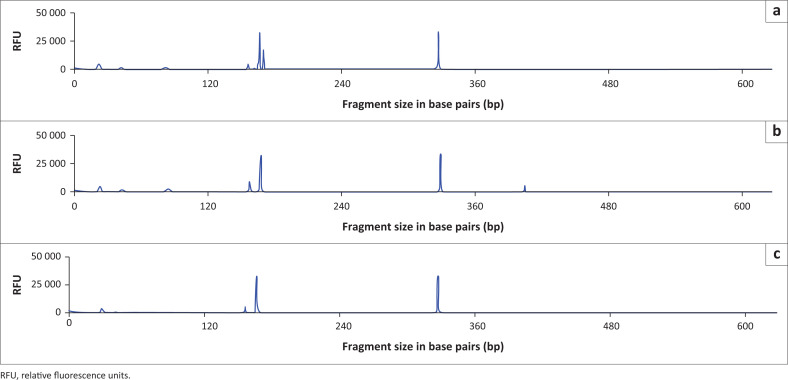
Observed capillary electropherograms of the *NPM1* and *FLT3* control samples (February 2020, Bloemfontein, Free State, South Africa). (a) *NPM1* positive control with an additional amplicon at 174 base pairs representing the 4 base pairs insertion, and wild-type *FLT3-*ITD at 330 base pairs. (b) *FLT3*-ITD positive control with an additional amplicon at 405 base pairs, representing the 75 base pairs internal tandem duplication and *NPM1* wild-type at 170 base pairs. (c) *NPM1* and *FLT3*-ITD negative control with expected amplicons at 170 base pairs and 330 base pairs.

The optimised *NPM1* and *FLT3*-ITD multiplex PCR was used to screen the 40 de novo AML patients for the respective mutations. Positive and negative controls, as well as a no-template control, were included in each subsequent run for quality control purposes. The *NPM1* and *FLT3*-ITD wild-type amplicon sizes were expected to be 170 base pairs (bp) and 330 bp. The *NPM1* positive samples were expected to have an amplicon at 170 bp for the wild-type and an additional amplicon at 174 bp. *FMS*-related tyrosine kinase 3-ITD positive samples were expected to have an amplicon at 330 bp and an additional amplicon larger than 330 bp. The allele ratio for the respective mutations in each patient was calculated by determining the area under the curve for the mutant to wild-type alleles, expressed as a percentage. The respective positive and negative controls yielded the expected amplicon sizes upon evaluation of the capillary electrophoresis results (Figure 1).

### Sanger sequencing of *NPM1* and *FLT3-*ITD amplicons

*NPM1* and *FLT3*-ITD control samples, as well as the amplicons from the 40 patient samples, were sequenced to confirm and validate the PCR results. Sequencing reactions for the *NPM1* and *FLT3*-ITD mutations were prepared separately using primers as listed above (primers for sequencing were not fluorescently labelled). The PCR products were purified using the ExoSAP-IT® Express PCR Product Clean-up (Affymetrix, Santa Clara, California, United States) as per the manufacturer’s instructions. Sanger sequencing reactions were prepared using the BigDye™ Terminator version 3.1 kit (Applied Biosystems, Foster City, California, United States) as per the manufacturer’s instructions. Briefly, the sequencing reaction consisted of 2 *µ*L Sequencing Reaction Mix (BigDye™ Terminator v3.1), 1 *µ*L Sequencing Buffer (BigDye™ Terminator v3.1), 5 *µ*L nuclease-free water, 1 *µ*L forward primer (*FLT3* or *NPM1*), 1 *µ*L reverse primer (*FLT3*-ITD or *NPM1*) and 1 *µ*L PCR product. The sequencing reaction was subjected to the following cycling conditions in a 2720 Thermal Cycler (Applied Biosystems, Foster City, California, United States): one cycle at 96 °C for 1 min, and 25 cycles at 96 °C for 10 s, 50 °C for 5 s, and 60 °C for 4 min. Sequence reactions were cleaned up with the Zymo Research DNA Sequencing Clean-up Kit™ (Promega Madison, Wisconsin, United States) as per the manufacturer’s instructions. Sequence raw reads were loaded onto the 3500 Genetic Analyser (Applied Biosystems, Foster City, California, United States). For comparison, sample sequences were aligned against *NPM1* (GenBank accession: NG_016018.1) and *FLT3*-ITD (GenBank accession: NG_007066.1) reference sequences retrieved from the National Center for Biotechnology Information using the Local Pairwise Sequence Alignment Algorithm software (Available at https://embnet.vital-it.ch/software/LALIGN_form.html). Sanger sequence analysis confirmed that the *NPM1* positive control had a 4 bp insertion (bases: CATG), and the *FLT3*-ITD positive control had a 75 bp ITD. The mutation-negative controls were compared to the respective reference sequences, and were found to be 100% similar.

### Data analysis

Fragment analysis electropherograms (Figure 1) were generated using the GeneMapper v6 software (Applied Biosystems, Foster City, California, United States), and displayed the size of the alleles obtained for the target PCR fragments (Applied Biosystems, Foster City, California, United States). *FLT3*-ITD allelic ratios were determined by calculating the ratio of the area under the curve of the *FLT3*-ITD mutant allele to the *FLT3* wild-type allele as displayed by GeneMapper.

The sequence was analysed using the Sequencing Analysis Program version 5.3.1 (Applied Biosystems, Foster City, California, United States) and Chromas version 2.6.6 (Technelysium Pty Ltd, Brisbane, Australia). Tables were generated using Microsoft Word 2016 (Microsoft, Redmond, Washington, United States). Patients’ demographic data and AML classification were summarised in table format. Ethnicity, defined as either black, white or mixed race, was based on patients’ self-proclaimed identity and accordingly documented.

## Results

The AML patients (FN1 to FN40) were all successfully screened with the *NPM1* and *FLT3*-ITD multiplex PCR assay. The AML patient cohort had a median age of 38.5 years (range 18–85 years) at presentation and the ratio of women to men was 1:1.2. The cohort included 31 black African patients, four white patients, and five patients of mixed ethnicity. Acute myeloid leukaemia subtypes in our patient cohort comprised of AML with recurrent genetic abnormalities, AML with myelodysplasia-related changes, and AML not otherwise specified (also referred to as CN-AML). Acute myeloid leukaemia with recurrent genetic abnormalities was most prevalent (57.5%; *n* = 23), of which acute promyelocytic leukaemia (APL) was most common (40.0%; *n* = 16). None of the patients had the *NPM1* mutation. *FMS*-related tyrosine kinase 3-ITD was present in 37.5% (6/16) of APL patients and in one (20.0%) of five AML patients with a t(8; 21) translocation (Table 1). Patients with an *FLT3*-ITD mutation had a median age of 30.8 years at presentation, and the majority of patients were black Africans (*n* = 5/7) (Table 2). Internal tandem duplication lengths of the *FLT3* mutations observed in this study ranged between 19 bp and 75 bp, and the *FLT3*-ITD allele ratios ranged between 17.5% and 94.9%.

**TABLE 1:** Classification of acute myeloid leukaemia patients’ *NPM1* and *FLT3*-ITD genotypes (August 2020, Bloemfontein, Free State, South Africa).

AML classification	*NPM1*wt/ *FLT3*-ITDwt		*NPM1*Δ/ *FLT3*-ITDΔ		*NPM1*wt/ *FLT3*-ITDΔ		*NPM1*Δ/ *FLT3*-ITDwt	
	*n*	%	*n*	%	*n*	%	*n*	%
**AML with recurrent genetic abnormalities**	16	40.0	0	0	7	17.5	0	0
Translocation t(8; 21)	4	10.0	0	0	1	2.5	0	0
Inversion 16	1	2.5	0	0	0	0	0	0
Translocation t(9; 22)	1	2.5	0	0	0	0	0	0
APL translocation t(15;17)	10	25.0	0	0	6	15.0	0	0
**AML with myelodysplasia-related changes**	3	7.5	0	0	0	0	0	0
**AML not otherwise specified (CN-AML)**	14	35.0	0	0	0	0	0	0
Minimal differentiation	1	2.5	0	0	0	0	0	0
Without maturation	3	7.5	0	0	0	0	0	0
With maturation	6	15.0	0	0	0	0	0	0
Myelomonocytic	4	10.0	0	0	0	0	0	0

AML, acute myeloid leukaemia; wt, wild-type; Δ, mutant; APL, acute promyelocytic leukaemia; ITD, internal tandem duplication; CN-AML, cytogenetically normal AML.

**TABLE 2:** Acute myeloid leukaemia patients positive for the *FLT3*-ITD mutation (August 2020, Bloemfontein, Free State, South Africa).

Patient	AML classification	Age (years)	Sex	Race	ITD length (bp)	Allele ratio (%)
FN14	APL t(15; 17)	25	M	ME	39	63.3
FN19	APL t(15; 17)	26	F	B	27	48.4
FN20	APL t(15; 17)	19	M	B	59	50.1
FN21	APL t(15; 17)	33	M	B	44	94.9
FN26	APL t(15; 17)	59	F	W	50	39.6
FN32	APL t(15; 17)	34	F	B	19	79.5
FN36	t(8; 21)	20	M	B	75	17.5

AML, acute myeloid leukaemia; APL, acute promyelocytic leukaemia; M, male; F, female; ME, mixed ethnicity; B, Black; W, White; ITD, internal tandem duplication; bp, base pairs.

## Discussion

Acute myeloid leukaemia is a genetically heterogeneous disease^2^ with numerous clinical and genetic factors influencing the final diagnosis. The recognition of additional molecular abnormalities, such as *NPM1* and *FLT3*-ITD in AML, has ensured that the disease biology is better understood and has allowed for improved risk stratification to be included in the diagnostic algorithms.^3,4^ This is the first study to investigate the presence of *NPM1* and *FLT3*-ITD in a central South African AML population. The *NPM1* was absent in this study population, while the *FLT3*-ITD mutation predominated in the APL subtype. Furthermore, the majority of patients that were positive for the *FLT3*-ITD mutation had long ITD lengths and allelic ratios above 50%.

It was notable that the median age of our patient cohort was 38.5 years. The global median age at presentation for AML is 67 years^18^ and, in comparison, the current patient cohort presented with the disease at a considerably younger age. This finding was similar to a previous South African study, where adult AML patients presented with the disease at a median age of 41 years.^15^ Most patients in this study were self-proclaimed Black Africans, and there was no predominant disease presentation based on sex. Two other African studies found a similar age distribution and sex ratio.^19,20^

The *FLT3*-ITD mutation was only detected in patients with AML with recurrent genetic abnormalities, of which APL predominated. Compared to a reported frequency of *FLT3*-ITD mutations in APL of up to 40%,^16^ our results were not surprising. However, *FLT3*-ITD mutations in AML with a t(8;21) translocation, detected in one of our patients, is thought to be uncommon, occurring in less than 10% of AML cases.^21^ The reported frequency of *FLT3*-ITD mutations in CN-AML in African populations varies significantly from 11% in South African patients to up to 34.6% in an Egyptian cohort, which is more in line with data from high-income countries.^9,15,22^ The absence of *FLT3*-ITD in CN-AML patients in our study was unexpected, but could likely be attributed to the small study cohort. We recommend that the *FLT3*-ITD frequency should be investigated in a larger central South African CN-AML population.

Internal tandem duplication lengths of the *FLT3* mutations observed in this study ranged between 19 bp and 75 bp and were found in the juxtamembrane domain of the gene. According to Liu et al.,^23^ longer ITD lengths (more than 39 bp) have been associated with a worse prognosis, the possible reason being that longer insertions in the juxtamembrane domain may cause more significant disruption of the domain’s auto-inhibitory function.^23^ In our study, 71.4% (5/7) of the patients with *FLT3*-ITD mutations had an ITD length of 39 bp or longer. Further investigations are needed to determine whether the high prevalence of *FLT3*-ITD mutations in our APL cohort confers a worse prognosis. This investigation would be justified considering that the majority (4 of 6) of APL patients with *FLT3*-ITD mutations had allele ratios above 50% and in other AML subgroups, allelic ratios above 50% are stratified as an adverse prognostic risk.^24^

An interesting observation was the unusually high number of APL cases among our AML cohort, which, according to the revised 4th edition of the *Classification of Tumours of Haemopoietic and Lymphoid Tissues,* only accounts for 5% – 8% of AML cases.^16^ Notably, eight of our APL patients presented within a single month. Several studies, including a South African study, have alluded to the association between APL clustering and seasonality.^25,26,27^ With such a small study cohort, one can only speculate that seasonality may have contributed and warrants further investigation.

Similar to a Sudanese study involving 100 AML patients,^14^ none of the patients in this study had an *NPM1* mutation. However, *NPM1* mutations were detected in 28.3% of Egyptian CN-AML patients and 7.5% of South African AML patients.^13,15^ The complete absence of the *NPM1* mutation in this study could be attributed to the low median age at disease presentation in the study cohort, especially considering that the frequency of *NPM1* mutations increases proportionally with age.^28^ In addition, *NPM1* mutations are more commonly associated with CN-AML,^16^ which accounted for only 35% (*n* = 14) of all cases in this study.^4,9^ The Cancer Genome Atlas Research Network reported that nearly 50% of all AML patients are CN-AML cases, and the observed prevalence of CN-AML in the current study was lower.^6^ The lower prevalence of CN-AML relative to AML with recurrent genetic abnormalities in our study, and, by association, the lower prevalence of these two mutations commonly linked with CN-AML, might be attributed to race, as Black patients were previously found to be more likely to have AML associated with cytogenetic abnormalities.^18,29^

### Limitations

This study was limited by a small sample population. In addition, the predominance of APL cases in our cohort may have contributed to a biased prevalence of the *FLT3-*ITD mutation. An association between phenotype and genotype was also not determined.

### Conclusion

The *FLT3*-ITD mutation was mainly found in APL cases at a similar prevalence as reported in the literature. High *FLT3*-ITD allele ratios and long ITD lengths predominated. No *NPM1* mutations were detected. The absence and lower frequency of *NPM1* and *FLT3-*ITD mutations could possibly be attributed to the low median age at presentation and the majority of patients presenting with AML with recurrent genetic abnormalities.
